# *Solenopsis sphaciotica* (Campanulaceae) a new species from Crete

**DOI:** 10.3897/phytokeys.271.175650

**Published:** 2026-03-12

**Authors:** Salvatore Cambria, Gianpietro Giusso Del Galdo, Pietro Minissale, Giuseppe Siracusa, Salvatore Brullo

**Affiliations:** 1 Department of Biological, Geological and Environmental Science, University of Catania, Via A. Longo 19, I - 95125 Catania, Italy University of Catania Catania Italy

**Keywords:** Lobelioideae, Mediterranean region, morphology, pollen, seed testa, *
Solenopsis
*, taxonomy

## Abstract

A new species of *Solenopsis* (Campanulaceae), *S. sphaciotica*, is described and illustrated from Crete, Greece. Morphologically, it shows close affinities with *S. gutermannii* by having a small size, annual habit and rosulate leaves. Although it was previously confused with *S. minuta* due to similarities in habit and some flower traits, several relevant morphological features clearly distinguish it from the latter. Furthermore, while it shares the annual life form, rosulate leaves and corolla shape with *S. antiphonitis*, it is also well differentiated from that species. This study provides a comprehensive examination of the species’ morphology, seed coat and pollen micro-sculpturing, ecology, distribution, conservation status and taxonomic relationships.

## Introduction

Herbarium research and field investigations concerning the genus *Solenopsis* on the island of Crete (Greece) confirmed that the most widespread populations in this region belong to *S. minuta* (L.) C.Presl, which is morphologically distinct from other species currently known in the rest of the Mediterranean (Crespo et al. 1998; Brullo et al. 2023a, 2023b, 2023c, 2024). More broadly, recent phylogenomic analyses across Campanulaceae have revealed substantial gene-tree conflict and reticulation, with implications for generic delimitation and species boundaries (Lin et al. 2025; Xu et al. 2025). Within *S. minuta*, Greuter et al. (1984) recognised two subspecies differentiated mainly by their perennial or annual life form. Specifically, the authors described subsp. minuta showing a perennial habit and subsp. *annua*, typically an annual plant, a character that varies amongst populations due to environmental influence. Our analysis shows that perennial and annual individuals do not seem to differ much in their vegetative and floral traits, except that the annual plants exhibit a therophytic habit. Another species morphologically well distinct from *S. minuta* is *S. laurentia* (L.) C.Presl, which, in Crete, is localised in the north-west of the island, growing in humid stands, especially near the coast, where it is linked to hygrophilous therophytic communities (Muer et al. 2024). According to Brullo et al. (2023a), the Cretan populations of this species must be referred to subsp. *laurentia*. Another very peculiar population of *Solenopsis*, morphologically differentiated from the other two aforesaid species, was observed in the south-western region of the island, near Hora Sfakia, in dripping rocks close to the coast. The individuals localised in this extremely specialised habitat, when kept in cultivation, maintain a small size even after several years of cultivation; it is not an environmental adaptation, but these characteristics are clearly genetically fixed. This population was also examined by Greuter et al. (1984), who highlighted that it is strikingly different from other populations of *S. minuta* present in the rest of the island, primarily owing to its extremely dwarfed habit. Initially, due to the extremely small size of these individuals, these authors provisionally attributed them to *Laurentia gasparrinii*, belonging to the cycle of *Solenopsis laurentia*; only later did they refer them to *S. minuta* subsp. *annua*. This small population also differs from other annual species with very similar habits, such as *S. antiphonitis* Hadjik. & Hand and *S. gutermannii* Brullo, Cambria, Costanzo & Giusso, in several morphological features, especially regarding the shape and size of vegetative and reproductive traits. Based on these data, we formally describe this population as a species new to science and name it *S. sphaciotica*.

## Materials and methods

Morphological investigations of *Solenopsis sphaciotica* utilised wild plants collected from the type locality in Crete and subsequently cultivated in the Botanical Garden of Catania (Italy). For comparative analyses, we examined fresh *S. minuta* material collected from several localities of Crete, in addition to living material of *S. antiphonitis* from the type locality in Cyprus and data provided by Brullo et al. (2025) for *S. gutermannii*. Living material of these species has also been cultivated for several years (2–3) in the Botanical Garden of Catania, under optimal conditions, without ecological stress, through the seeds produced by the plants themselves. The cultivated plants were isolated to avoid possible hybridisation. Qualitative and quantitative morphological features with diagnostic value in the genus *Solenopsis* were measured and scored on at least ten cultivated individual plants. In addition, herbarium specimens kept in B, CAT, CBH, FI, G, JE, P, PAL-GREUTER, W, WAG and WU (acronyms according to Thiers (2025)), were also consulted online. The material was observed under a Zeiss Stemi SV 11 Apo stereomicroscope at 6–66× magnification. Comparative diagnostic features of the investigated taxa are listed in Table 1. Electron micrographs (SEM) of seeds were obtained under a Zeiss EVO LS10 scanning electron microscope at an accelerating voltage of 10 kV. In particular, 10 seeds were directly mounted on to aluminium stubs with double adhesive tape and coated with gold prior to observation. The terminology describing the seed testa sculpturing mainly followed Barthlott (1981, 1984) and Gontcharova et al. (2009). Pollen morphology was examined using a scanning electron microscope (SEM) Zeiss EVO LS10. Dried pollen was mounted on stubs without any preparation and terminology follows Walker and Doyle (1975), Punt et al. (1994, 2007) and Hesse et al. (2009). Pollen material was obtained from living plants cultivated in the Botanical Garden of Catania (Italy). The vouchers are deposited in the Herbarium of the University of Catania (CAT).

**Table 1.** Main diagnostic morphological characters of *Solenopsis sphaciotica* and allied species.

Taxa	*S. sphaciotica*	*S. minuta*	*S. gutermannii*	*S. antiphonitis*
**Characters**				
Life form	annual	annual to perennial	annual	annual
Habit	acaulescent	acaulescent	acaulescent to briefly scapose	acaulescent
Leaf rosette diameter (cm)	0.4–3(4)	(2–)2.5–6	1.2–2	1.5–5 (2–2.5)
Leaf length (mm)	2–17	7–30	4–12	6–18 (4–15)
Leaf margin glands	no	yes	no	yes
Leaf blade size (mm)	1–7 × 0.5–4	3–14 × 2–7	2–5.5 × 1.2–3	2–8 × 1.5–5
Petiole length (mm)	2–12	3–13(20)	2–8	4–11 (1.5–7)
Bracteole size (mm)	1.1–2.2 × 0.1–0.3	(2–)2.5–4.5 × 0.3–0.7	1.5–2.7 × 0.2–0.3	1.8–2.5 (2.2–5 × 0.25–1)
Bracteole hairs	1 apical and 1–3 lateral	1 apical and 2–4 lateral	1 apical and 2–4 lateral	0–1 lateral
Bracteole glands	2–3 per side	1–2 per side	1–2 per side	(1)2–4 per side (1 apical and 2–3 per side)
Floral pedicel (mm)	10–35	20–50	15–45	14–40(55) (2.5–3)
Calyx length (mm)	1.1–2	3.5–4.5(–5)	2.5–3.3	2–2.5
Calyx lobes length (mm)	1–1.1	(2–)2.5–3.5	1.8–2	1.3–1.7 (1.7–2.2)
Corolla length (mm)	4–5	7–10	6–7	5–6.5 (6)
Corolla tube length (mm)	1.8–2	2.5–3.5	2–2.5	3–3.5
Lobes upper lip colour	lilac	bluish-lilac	bluish-lilac	pale lilac to white-lilac
Lobes lower lip marginal colour	lilac, well distinct	bluish-lilac, well distinct	bluish-lilac, well distinct	pale lilac to white-lilac, gradient
Lobes upper lip size (mm)	1.7–2.3 × 0.8–0.9	3–4.2 × 1.2–1.4	2.5–2.7 × 1.0–1.2	2.7–2.9 × 1.4–1.5 (2.5–3.2 × 1.2–1.3)
Lobes upper lip papillae	no	yes	no	yes
Lower lip length (mm)	2.5–3.5	5–6	3.7–4.2	3.4–3.7 (3.2–3.5)
Lower lip lobes size (mm)	1.5–1.7 × 1.4–2	2.5–3.5 × 2.2–3.2	1.8–2.3 × 1.8–2.2	2.3–3.2 × 2.3–2.6 (1.8–2.3 × 1.6–2)
Lower lip papillae	up to lobe base	up to lobe middle	up to lobe base	up to lobe apex
Lower lip papillae length (mm)	0.06–0.12	0.05–0.4	0.02–0.2	0.13–0.25(0.38) (0.1–0.48)
Staminal filament length (mm)	2–2.5	3–5	3–3.5	2.5–3
Anther tube length (mm)	0.8–1	1.1–1.3	1.0–1.1	0.8–1
Anther tube basal papillae	no	no	no	yes, cylindrical and flat
Anther tube dorsal hairiness	yes, few apical	yes, few apical	yes, few apical	yes, up to the base
Style length (mm)	3–3.2	3.5–4.0	3–3.5	3.5–3.6 (3.2)
Capsule length (mm)	1.7–1.8	2.5	1.8–2.0	2–3
Capsule surface	smooth	smooth	papillose	smooth
Seeds (mm)	0.32–0.36 × 0.16–0.18	0.48–0.50 × 0.24–0.25	0.36–0.40 × 0.20–0.25	(0.33)0.36–0.38 × (0.2)0.23–0.25

## Taxonomic treatment

### Solenopsis sphaciotica

Taxon classificationPlantaeHymenopteraFormicidae

Cambria, Giusso, Miniss. & Brullo sp. nov.

9AF97E46-2ECE-5075-8268-3D64121E16C9

urn:lsid:ipni.org:names:77377650-1

Figs 1, 2, 3

#### Type.

Greece • Crete, along the road west of Hora Sfakion on carbonate dripping rocks, 35°12'14.90"N, 24°7'27.19"E, 53 m alt., 28 May 2022, *S. Cambria s.n*. (holotype CAT!; isotypes CAT! FI!).

**Figure 1.**
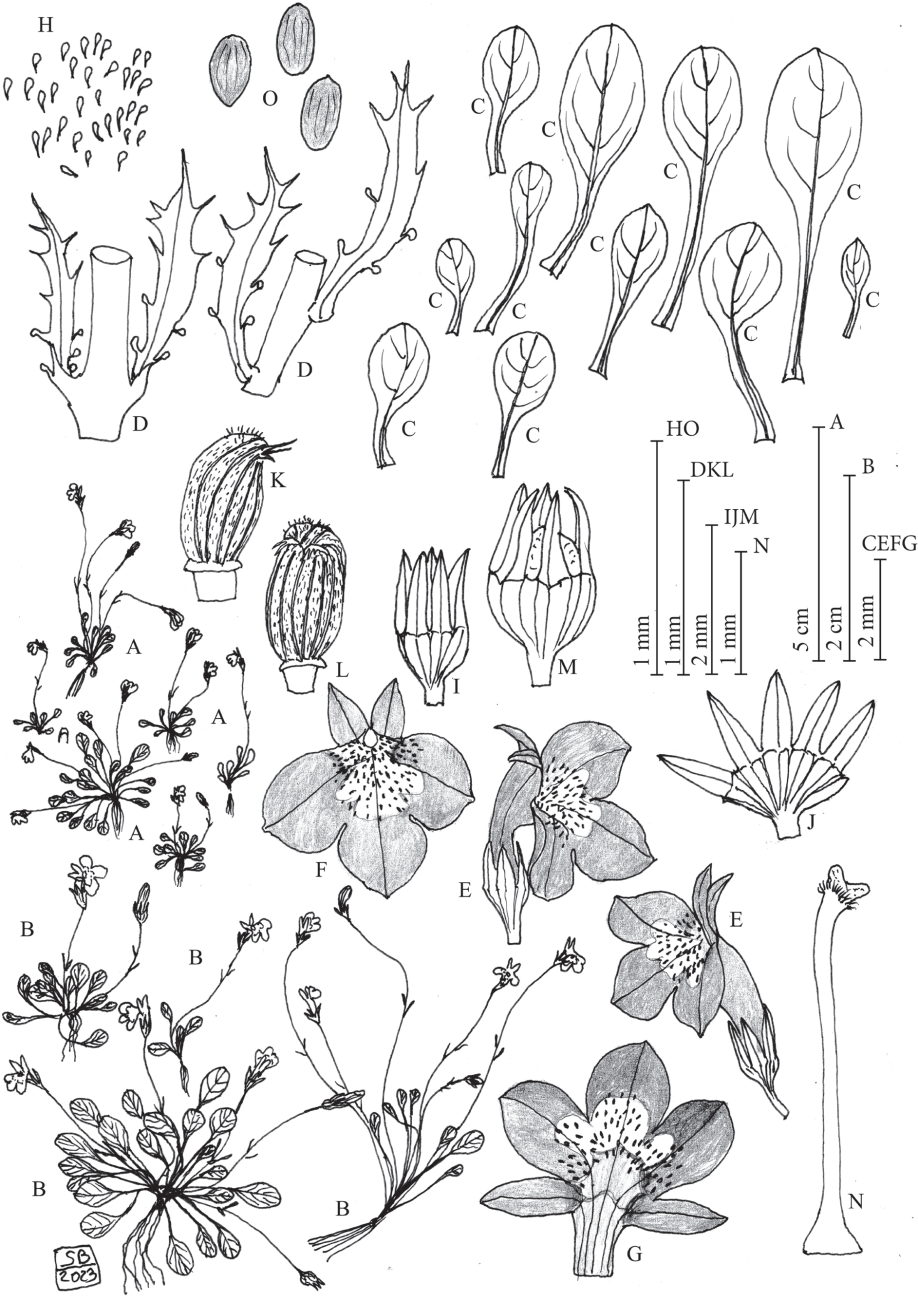
*Solenopsis sphaciotica* from the type locality. **A**. Habit; **B**. Habit magnification (× 2); **C**. Leaves; **D**. Bracteoles; **E**. Flower (lateral view); **F**. Flower (frontal view); **G**. Open corolla; **H**. Corolla papillae; **I**. Calyx; **J**. Open calyx; **K**. Anthers (lateral view); **L**. Anthers (frontal view); **M**. Calyx with capsule; **N**. Style and stigma; **O**. Seeds. Drawing: S. Brullo.

#### Diagnosis.

The new species is similar to *Solenopsis minuta* in having rosulate leaves, bracteole indumentum and corolla colour; however, it differs in having a much reduced annual habit with smaller morphological characters throughout, a more reduced leaf rosette, leaves without margin glands, smaller bracteoles, shorter calyx (1.1–2 mm long), paler and much smaller corolla (4–5 mm), up to 0.12 mm long papillae, occurring only at the base of the lower lip, shorter anther tube, staminal filament and style and smaller capsule and seeds.

**Figure 2.**
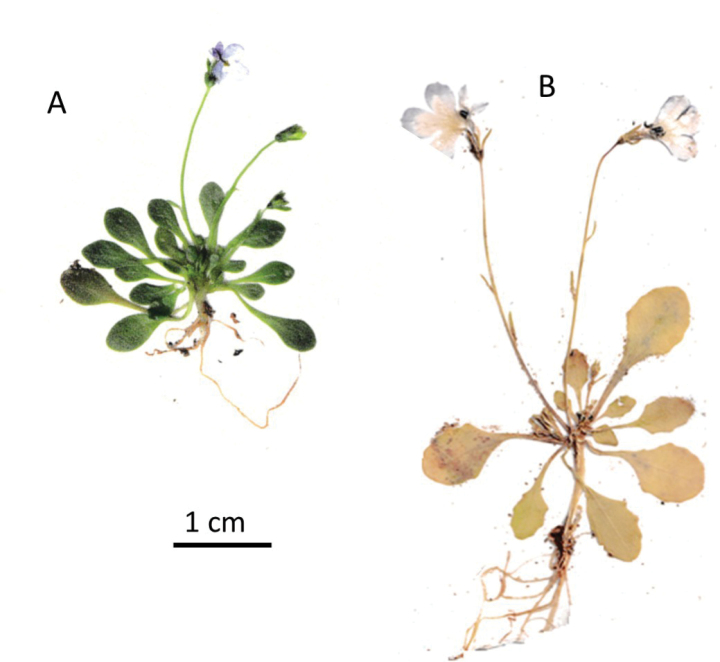
Habit of living plants of *Solenopsis sphaciotica* (**A**) and of *S. minuta* (**B**).

#### Description.

Annual herb, acaulescent or briefly scapose, usually rosulate, 1.2–2 cm in diameter, provided with fibrous slender roots. Leaves 4–12 mm long, spathulate to oblanceolate, with blade entire to weakly crenate, glabrous, without glands, 2–5.5 × 1.2–3 mm, with petiole 2–8 mm long. Floral pedicels 15–45 mm, with 2 bracteoles, 1.5–2.7 mm long, 0.2–0.3 mm wide, with 4–8 hairs in the upper half, of which 1 apical and 2–4 per side, stipulated glands 1–2 per side in the lower half. Calyx 2.5–3.3 mm long, with linear-lanceolate lobes, 1.8–2 mm long, glabrous. Corolla 4–5 mm long, bilabiate, with tube pale lilac, 2–2.5 mm long, 0.6–0.7 mm in diameter; upper lip with 2 lobes lanceolate, 2.5–2.7 mm long, 1.0–1.2 mm wide, bluish-lilac, acute at apex, without glands; lower lip trilobed, 4.5–5.5 mm long, yellowish at the base, lobes widely ovate and apiculate at the apex, 1.8–2.3 × 1.8–2.2 mm, widely edged in bluish-lilac and irregularly white in the central part until the base, covered by dense papillae up to the base of the lobes, 0.02–0.2 mm long. Stamen filaments free, 3–3.5 mm long, anthers violet, connate into a tube 1.0–1.1 mm long, wholly encapsulating the stigma; the two lower anthers are smaller, without papillae at basis, each appendiculate at the top with a tuft of hairs, closing a narrow fissure; the three upper anthers are curved, hairy dorsally. Ovary fused with the calyx tube; style whitish, 3.0–3.5 mm long; stigma pale lilac, bifid, papillate, with a ring of hairs just under the base. Capsule papillose, 1.8–2 mm long. Seeds ellipsoid, brownish shining, 0.36–0.40 × 0.18–0.20 mm.

**Figure 3.**
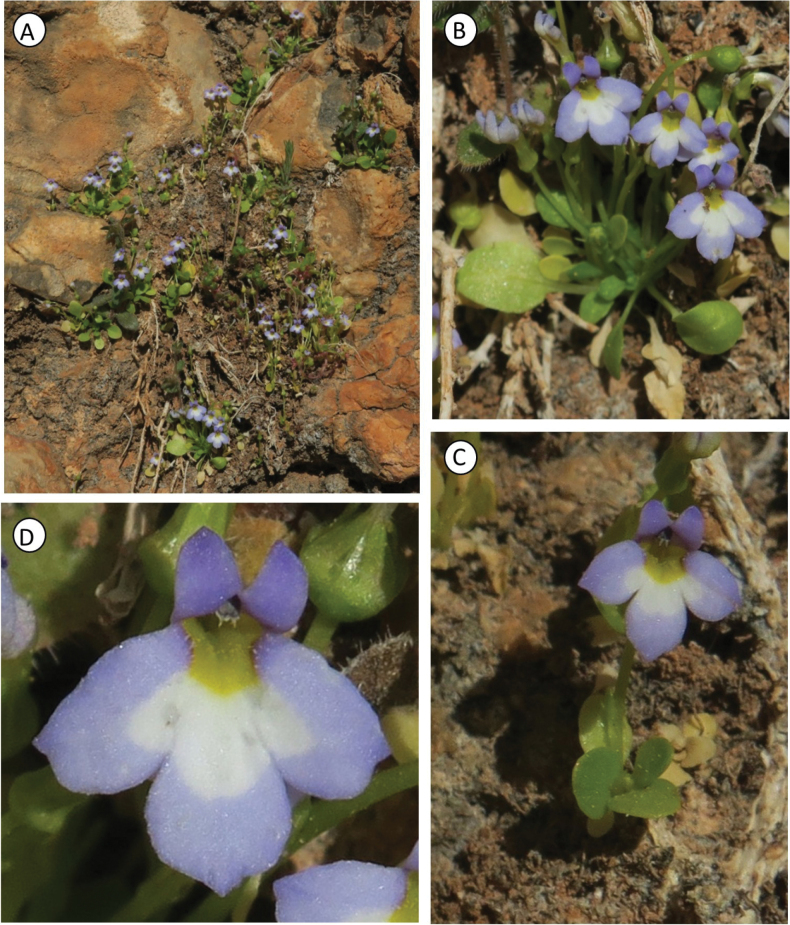
Phenological features of *Solenopsis sphaciotica*. **A**. Natural habitat (Crete); **B, C**. Habit; **D**. Flower (frontal view). Photos by S. Cambria.

#### Etymology.

The specific epithet, *sphaciotica*, refers to Hora Sfakion, the locality where the new species grows.

#### Phenology.

It flowers and fruits from April until early June, based on field observations and cultivated plants in the Botanical Gardens of Catania.

#### Distribution, habitat and ecology.

**Figure 4.**
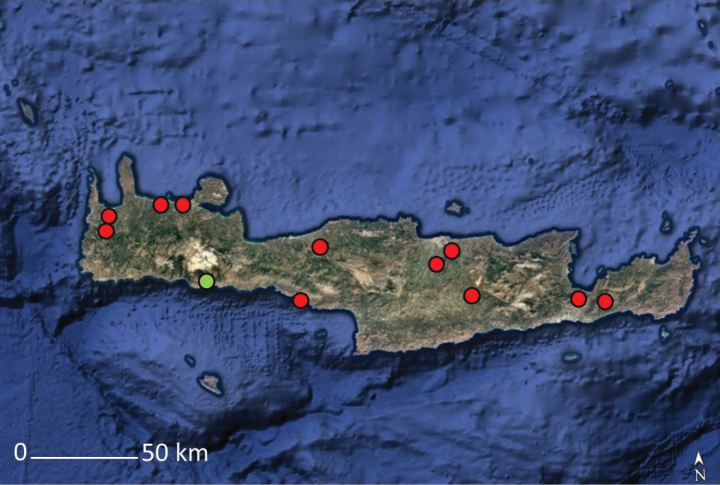
Distribution map of *Solenopsis sphaciotica* from Crete (green dot) and *S. minuta* from Crete (red dot), based on herbarium material.

*Solenopsis sphaciotica*, a very small annual hygrophyte, occurs in a single locality, near Hora Sfakion (Crete), where it is localised in temporarily dripping rocky walls covered by a layer of red soil rich in clay component (Fig. 4). In this wet microhabitat, it flowers during the spring together with other small microphytes such as *Campanula erinus* L., *Centaurium tenuiflorum* (Hoffmanns. & Link) Fritsch and *Sedum rubens* L., on a relatively sparse moss carpet dominated by *Gymnostomum calcareum* Nees & Hornsch. var. *calcareum*.

#### Conservation status.

The species is currently known from a single small population spanning less than 10 m^2^. No more than 100 individuals have been counted. The population is localised within the SAC (Special Area of Conservation) “Lefka Ori Kai Paraktia Zoni” (code GR4340008), representing a wide site of significant naturalistic interest. Consequently, any random natural or human-induced event could destroy the site and the entire population. Therefore, according to IUCN criteria (IUCN 2025), it is proposed to treat *S. sphaciotica* as critically endangered (CR), based on the B2a+D criteria.

#### Pollen grains micromorphology.

**Figure 5.**
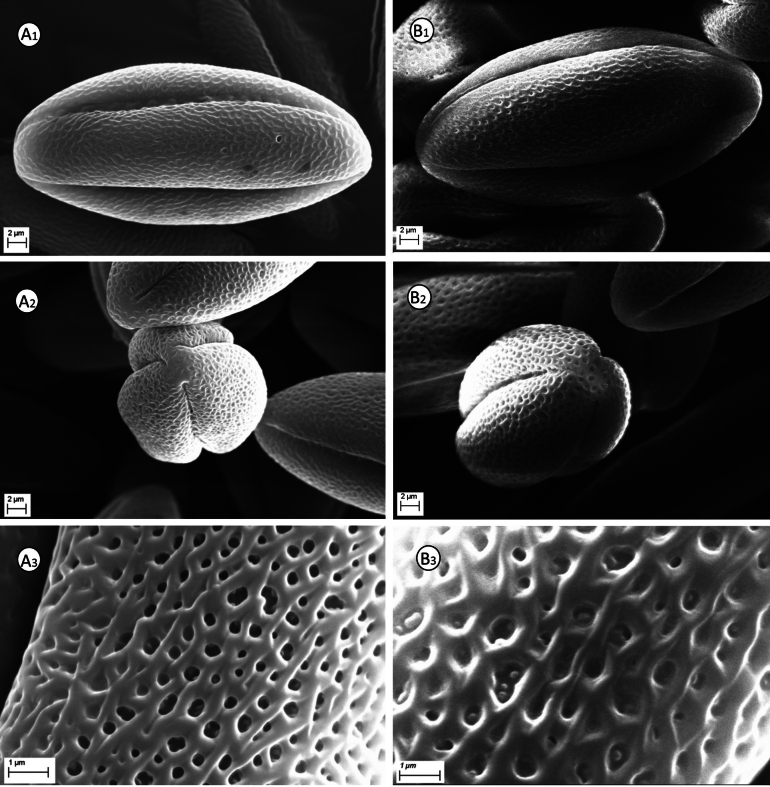
SEM micrographs of pollen grains of *Solenopsis sphaciotica* (**A**) and *S. minuta*. (**B**). **1**. Pollen equatorial view (× 2800); **2**. Pollen polar view (× 2800); **3**. Sexine ornamentation (× 12000). Images made by G. Siracusa.

SEM investigations carried out on dried pollen grains of *Solenopsis sphaciotica* revealed that they are, as well as all other previously studied species (Dunbar 1975; Brullo et al. 2023a, 2023c), 3-colporate with a perplorate shape. In particular, the pollen is ellipsoid, measuring 37.0–38.0 μm in length and 14.5–18.0 μm in width (Fig. 5A), with loosely reticulate sexine, characterised by slightly convex and irregularly anastomosed branched lirae, usually not overlapping, with a thickness of 0.2–0.5 μm, delineating irregular lumina 0.6–1.5 μm in diameter. Concerning the pollen grains of *S. minuta*, they are ellipsoid-fusiform, shorter, measuring 33.5–37.0 μm in length and 17.2–17.8 μm in width (Fig. 5B), with different sexine characterised by markedly convex and irregularly anastomosed branched lirae, clearly overlapping, with a thickness of 0.1–0.2 μm, delineating irregular lumina 0.23–0.6 μm in diameter.

#### Seed micromorphology.

**Figure 6.**
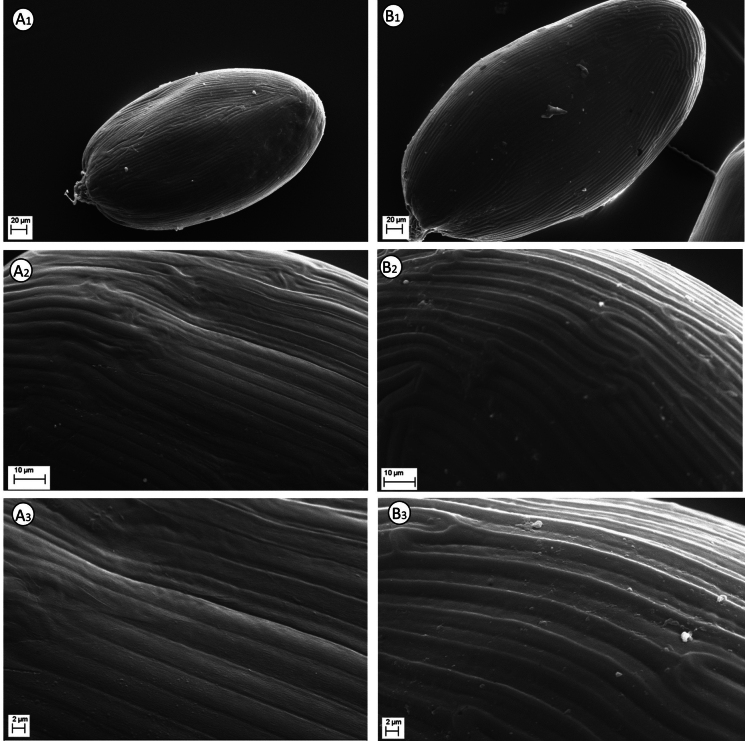
SEM micrographs of seeds of *Solenopsis sphaciotica* (**A**) and *S. minuta* (**B**). **1**. Whole seed (× 250); **2**. Detailed seed testa (× 1000); **3**. Detailed seed testa (×2 000). Images made by G. Siracusa.

The ornamentations of the seed coat surface in the Campanulaceae subfamily Lobelioideae hold remarkable diagnostic value and phylogenetic significance (Murata 1992, 1995; Haridasan and Mukherjee 1993; Serra and Crespo 1997; Crespo et al. 1998). Previously, the seed coat in *Solenopsis* was investigated by Serra and Crespo (1997), Crespo et al. (1998) and Brullo et al. (2013, 2023a, 2023b, 2023c), who emphasised that the sculptures are quite similar across all species. In particular, our SEM investigation carried out on *S. sphaciotica* highlights that the seeds (Fig. 6A) typically have an elliptical shape and are slightly rounded at the extremities, with a size of 0.31–0.35 × 0.18–0.20 mm. Regarding the seed testa, the cells show slightly convex periclinal walls, 3.2–5 μm wide, with incised anticlinal walls 0.4–0.5 μm wide (Fig. 6: A2, A3). In *S. minuta*, the seeds (Fig. 6B) typically have an oblong shape and are rounded at the extremities, with a size of 0.36–0.45 × 0.21–0.24 mm. Concerning the seed testa, the cells show raised and narrower periclinal walls, 2.8–3.0 μm wide, with flat and well-developed anticlinal walls, 2.0–2.8 μm wide (Figs 6: B2, B3).

#### Additional specimens examined of *Solenopsis sphaciotica* (Paratypes).

Greece • Crete, Griechland, W-Kreta, Ep. Sfakia, Südküste bei Chora Sfakion, Felsen an der Straβe nach Anopolis, 4 April 1980, *H. Kalheber 80-698* (PAL-GREUTER 35218!) sub *Laurentia gasparrinii*; • Crete, along the road West of Hora Sfakion on carbonate dripping rocks, 35°12'14.90"N, 24°7'27.19"E, 53 m alt., 15 May 2023, cultivated material, *S. Cambria & S. Brullo s.n*. (CAT!).

#### Additional specimens examined of *Solenopsis minuta*.

See Brullo et al. (2025).

#### Additional specimens examined of *Solenopsis antiphonitis*.

See Brullo et al. (2025).

#### Additional specimens examined of *Solenopsis gutermannii*.

See Brullo et al. (2025).

## Discussion

Due to its annual life-form and several morphological traits, such as leaves in basal rosettes, flower pedicels inserted at the base and divaricate and bicoloured corolla, *Solenopsis sphaciotica* shows close affinities with other eastern Mediterranean species. Specifically, it shows similarities with the annual form of *S. minuta* from Crete, *S. antiphonitis* from Cyprus and *S. gutermannii* from Kefalonia Island. However, *S. sphaciotica* differs significantly from the above mentioned species in having calyx 1.1–2 mm long (vs. 2–5 mm), corolla 4–5 mm long (vs. 5–10 mm), with upper lip lobes 1.7–2.3 mm long (vs. 2.5–4.2) and lower lip lobes 1.5–1.7 mm long (vs. 1.8–3.5), showing a definite lilac colour at the margin (vs. other different colours), flower papillae up to 0.12 mm long (vs. up to 0.2–0.4 mm), staminal filament 2–2.5 mm long (vs. 2.5–5 mm), capsule 1.7–1.8 mm long (vs. 1.8–3 mm), seeds 0.32–0.36 mm long (vs. 0.36–0.50 mm). In particular, *S. sphaciotica* differs from *S. minuta* also in terms of the size of the seeds, which, in the latter, are clearly larger (0.48–0.50 mm vs. 0.32–0.36) with different seed head decorations, as well as in terms of the pollen, which is smaller (33.5–37.0 μm vs. 37.0–38.0 μm) and with a different sexine. The other differences between these species are reported in Table 1, where all the morphological characters are compared across species. *S. sphaciotica* shows greater affinities with *S. antiphonitis* primarily due to its small flower size; however, it is readily distinguished by having leaves without glands at the margin, much shorter calyx and corolla, lilac corolla lobes with a distinct margin in the lower lip (vs. pale lilac to white-lilac, shaded in the lower lip lobes), absent papillae in the upper lip (vs. occurring in the upper lip), papillae only at the base of the lower lip (vs. papillae up to the apex), anther tube without basal papillae (vs. two tufts of papillae) and smaller capsules and seeds. Ecologically, *S. sphaciotica* and *S. antiphonitis* share similar requirements, both being localised on small rocky walls temporarily affected by dripping water. In particular, *S. sphaciotica* appears to be more thermophilic than *S. antiphonitis*, which prefers a more mesic habitat, affected by very shaded conditions and higher elevations between 200 and 300 m. Notably, *S. minuta* and *S. gutermannii* always grow on more or less flat surfaces with flooded soils or remaining humid throughout the year, except in cases where water stress occurs due to very dry summers that cause the death of the individuals and the new generations necessarily reproduce only from seeds.. Therefore, the two species colonise habitats clearly very distinct, justifying their different life-forms and different size of their vegetative and floral structures. These different ecological adaptations probably influenced the speciation processes that occurred during their evolution. Additionally, in Crete, *S. minuta* is widespread throughout the island with distinct ecological requirements and phenology (late April until September), while *S. sphaciotica* shows a punctiform and isolated range and very different ecological requirements and phenology (April until early June). A similar event also occurred on the island of Cyprus where the most widespread *Solenopsis* species is *S. meikleana* Brullo et al., (cf. Brullo et al. 2023b), with a perennial habit and dimensions similar to those of *S. minuta*, which is replaced in temporarily humid microhabitats by *S. antiphonitis*, an annual species of small size, which is very close taxomically to *S. sphaciotica*.

### Key to the taxa belonging to the *Solenopsis* genus

Based on current knowledge of the genus *Solenopsis* (Crespo et al. 1998; Brullo et al. 2013, 2023a, 2023b, 2023c, 2024, 2025), an analytical key is elaborated to accommodate all taxa recognised or quoted in the recent literature (POWO 2025). Regarding *S. laurentia* subspecies, refer to Brullo et al. (2023a) and for those of *S. bivonae*, refer to Brullo et al. (2023b).

**Table:** 

1	Plant erect to subacaulescent, with leaves and flower pedicels all or partially inserted on the stem	**2**
–	Plant stemless (rarely subcaulescent); leaves all rosulate and flower pedicels inserted at the base	**4**
2	Corolla with lobes always patent, lower lip widely white in the central part and lilac to bluish at the margins	***S. bicolor***
–	Corolla with lobes connivent or slightly divaricate, uniformly coloured	**3**
3	Flower pedicels provided with one bracteole; calyx 2–2.5 mm long; corolla white, 3–3.5 mm long	***S. mothiana***
–	Flower pedicels usually provided with two bracteoles; calyx 2.5–5 mm long; corolla blue-lilac, 3.5–6 mm long	***S. laurentia***
4	Plant always annual	**5**
–	Plant perennial (sometimes annual)	**7**
5	Calyx 1.1–2 mm long; corolla 4–5 mm long, with lower lip lobes 1.5–1.7 mm long; staminal filament 2–2.5 mm long; capsule 1.7–1.8 mm long; seeds 0.32–0.36 mm long	***S. sphaciotica***
–	Calyx 2–3.3 mm long; corolla 5–7 mm long, with lower lip lobes 1.8–3.2 mm long long; staminal filament 2–3.5 mm long; capsule 1.8–3 mm long; seeds 0.36–0.40 mm long	**6**
6	Leaf blade without glands at the margin; calyx 2.5–3.3 mm long; corolla bluish-lilac, with tube 2–2.5 mm long and lower lip with papillae at the base; capsule 1.8–2 mm long	***S. gutermannii***
–	Leaf blade with glands at the margin; calyx 2–2.5 mm long; corolla lilac to white-lilac with tube 3–3.5 mm long and lower lip with papillae up to lobe apex; capsule 2–3 mm long	***S. antiphonitis***
7	Corolla lips uniformly dark blue-lilac (rarely white near the throat)	**8**
–	Corolla lips bluish-lilac, white in central part of lower lip	**9**
8	Bracteoles with 4–10 hairs on each side; corolla 13–16 mm long, with upper lip acute, 2.5–3.3 mm long; papillae covering only the throat of the lower lip; seeds 0.50–0.52 mm long and 0.30–0.32 mm wide	***S. bacchettae***
–	Bracteoles with 0–4 hairs on each side; corolla 6.3–6.5 mm long, with upper lip obtuse, 5–7 mm long; papillae covering the lower lip up to the lobe base; seeds 0.40–0.44 mm long and 0.24 mm wide	***S. corriasii***
9	Leaves hairy	**10**
–	Leaves glabrous or rarely subglabrous	**12**
10	Leaves irregularly lobate, with petiole 15–20 mm long; calyx 3.5–4.5 mm long; corolla 7–9 mm long; anther tube dorsally hairy; capsule 1–2 mm long	***S. balearica***
–	Leaves entire or slightly crenate, with petiole 2–15 mm long; calyx 2.4–3 mm long; corolla 4–4.5 mm long; anther tube dorsally glabrous; capsule 2–2.5 mm long	**11**
11	Bracteoles with 3–6 lateral hairs; calyx 2.5–3 mm long; corolla with upper lip 1.4–1.5 mm long and lower lip ovate and obtuse; papillae 2–4 in the basal part of lower lip, 0.18–0.3 mm long; anther tube without basal papillae	***S. corsica***
–	Bracteoles with 7–12 lateral hairs; calyx 2.4–2.5 mm long; corolla with upper lip 1.8–2 mm long and lower lip widely ovate and mucronate; papillae densely covering the throat and the basal part of the lower lip, 0.08–0.15 mm long; anther tube with two tufts of basal papillae	***S. limbarae***
12	Leaves max. 30 mm long, with blade max. 14 mm long and 7 mm wide; flower pedicel max. 50 mm long; corolla with upper lip 2.5–2.7 mm long	***S. minuta***
–	Leaves up to 100 mm long, with blade up to 40 mm long and 15 mm wide; flower pedicel up to 120 mm long; corolla with upper lip 3–6 mm long	**13**
13	Corolla lips pale blue to pale violet; corolla throat uniformly greenish-yellow; lower lip of corolla with lobes oblong, anther tube 1–1.5 mm long; style 3.5–4 mm long	***S. meikleana***
–	Corolla lips bluish-lilac; corolla throat yellowish to greenish bordered with brown; lower lip of corolla with lobes ovate; anther tube 1.4–1.9 mm long; style 4–7 mm long	***S. bivonae***

## Supplementary Material

XML Treatment for Solenopsis sphaciotica

## Additional information

### Use of AI

No use of AI was reported.

### Author ORCIDs

Salvatore Cambria https://orcid.org/0000-0002-3828-1552

Gianpietro Giusso Del Galdo https://orcid.org/0000-0003-4719-3711

Pietro Minissale https://orcid.org/0000-0002-4047-4169

Giuseppe Siracusa https://orcid.org/0000-0002-1989-6906

Salvatore Brullo https://orcid.org/0000-0003-2568-7278

### Data availability

All of the data that support the findings of this study are available in the main.
